# P-740. Prevalence of Methicillin-Resistant Staphylococcus Aureus and Pseudomonas in Wounds of Patients with Substance Use Disorder

**DOI:** 10.1093/ofid/ofae631.936

**Published:** 2025-01-29

**Authors:** Jerry Yang, Edward C Traver, Chih Chun Tung, Anthony Harris, Jonathan Baghdadi

**Affiliations:** University of Maryland Medical System, Baltimore, Maryland; University of Maryland School of Medicine, Baltimore, MD; University of Maryland School of Pharmacy, Baltimore, Maryland; University of Maryland School of Medicine, Baltimore, MD; University of Maryland School of Medicine, Baltimore, MD

## Abstract

**Background:**

Wound infections among patients with substance use disorder are frequently treated with empiric antibiotic coverage for Methicillin-resistant Staphylococcus aureus (MRSA) and Pseudomonas aeruginosa. To guide appropriate empiric antibiotics, we evaluated the prevalence of these pathogens in the wounds of patients with substance use disorder using a large database.

**Table 1.**
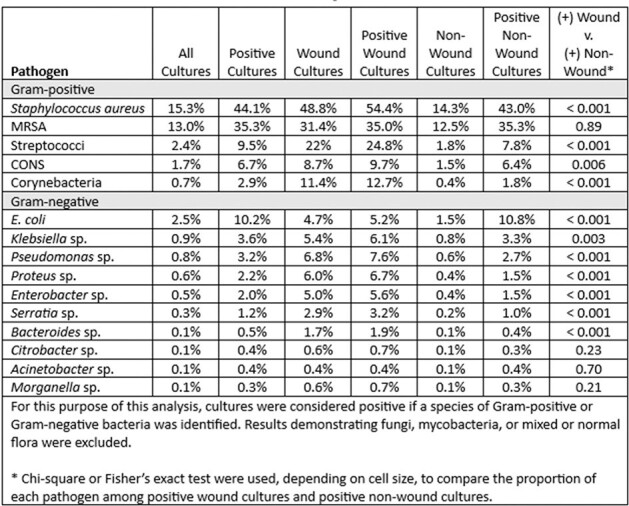
Prevalence of Pathogens in Wound and Non-Wound Clinical Cultures from Patients with Substance Use Disorder in a Large Administrative Database

**Methods:**

Encounters between January 2019 and March 2021 for patients with substance use disorder in the Premier Healthcare Database were identified by current or previous diagnosis code for opioid use, current or previous prescription of medications for opioid use disorder, or a diagnosis of Hepatitis C under the age of 55. Clinical cultures from any specimen besides stool were included.

**Results:**

Overall, 18,797 clinical cultures were collected during 10,720 encounters for patients with substance use disorder at 119 hospitals. 516 cultures from 378 encounters at 23 hospitals were explicitly labeled as being collected from wounds. Results from wound and non-wound clinical cultures are provided in Table 1. 426 wound cultures (83% of 516) demonstrated Gram-positive organisms, 230 (45%) demonstrated Gram-negative organisms, and 278 (54%) were polymicrobial. The most common organism identified was Staphylococcus aureus (n=252, 49% of wound cultures), 64% of which was MRSA. Pseudomonas was identified in 35 wound cultures (7%).

**Conclusion:**

Wound cultures from patients with substance use disorder are frequently polymicrobial. Though MRSA is extremely common, Pseudomonas is rare. Empiric antibiotic coverage of Pseudomonas should not be automatic.

**Disclosures:**

**Anthony Harris, MD, MPH**, Innoviva: Advisor/Consultant|UpToDate: Infection Control Editor

